# One mutation, divergent journeys: expanding the clinical spectrum of homozygous *SAMHD1* deficiency in childhood

**DOI:** 10.1093/rheumatology/keaf695

**Published:** 2026-01-06

**Authors:** Hülya Ercan Emreol, Dilara Ünal, Deniz Nazire Cagdas Ayvaz, Yelda Bilginer, Seza Özen

**Affiliations:** Department of Paediatrics, Division of Rheumatology, Hacettepe University Faculty of Medicine, Ankara, Turkey; Department of Paediatrics, Division of Rheumatology, Hacettepe University Faculty of Medicine, Ankara, Turkey; Department of Paediatrics, Division of Immunology, Hacettepe University Faculty of Medicine, Ankara, Turkey; Department of Paediatrics, Division of Rheumatology, Hacettepe University Faculty of Medicine, Ankara, Turkey; Department of Paediatrics, Division of Rheumatology, Hacettepe University Faculty of Medicine, Ankara, Turkey

**Keywords:** SAMHD1 deficiency, Aicardi-Goutières syndrome type 5, type I interferonopathy, paediatric autoinflammatory disease, JAK inhibitor, phenotypic heterogeneity, panniculitis, lupus-like syndrome, myositis, calcinosis

## Abstract

**Objectives:**

Homozygous loss-of-function mutations in *SAMHD1* classically cause Aicardi–Goutières syndrome type 5 (AGS5), characterized by neuroinflammation and intracranial calcifications. Increasing evidence suggests a broader clinical spectrum. We aimed to describe the phenotypic heterogeneity associated with a single homozygous *SAMHD1* variant in paediatric patients and to highlight diagnostic and therapeutic implications.

**Methods:**

We retrospectively reviewed three paediatric patients evaluated at a tertiary centre who carried the same homozygous *SAMHD1* missense variant (c.625G>A; p. Gly209Ser). Clinical features, laboratory findings, imaging results, genetic analyses, treatments and longitudinal responses were extracted from medical records. Whole-exome sequencing confirmed the pathogenic variant in all patients.

**Results:**

Despite sharing an identical homozygous *SAMHD1* mutation, the patients exhibited markedly divergent phenotypes: a chronic myopathy-dominant presentation without central nervous system involvement, a classical interferonopathy with panniculitis and intracranial calcifications and a lupus-like connective tissue disease phenotype with calcinosis and vasculopathy. Neuroimaging findings ranged from normal to classical AGS features. All patients received Janus kinase (JAK) inhibitors, predominantly tofacitinib, resulting in partial to sustained clinical improvement. Disease flares consistently occurred during treatment interruptions, emphasizing the importance of continuous therapy.

**Conclusions:**

Homozygous *SAMHD1* deficiency demonstrates striking phenotypic heterogeneity in childhood, extending beyond classical AGS. *SAMHD1* mutations should be considered in children with unexplained systemic inflammation, even in the absence of typical neuroimaging findings. Clinical responses to JAK inhibition across diverse phenotypes support a shared interferon-driven pathogenesis and highlight the value of early genetic diagnosis to guide targeted therapy.

Rheumatology key messages

- Homozygous SAMHD1 deficiency causes diverse paediatric phenotypes, including myopathy, panniculitis and lupus-like disease.
- Consider SAMHD1 deficiency in children with systemic inflammation despite absent classical AGS neuroimaging findings.
- JAK inhibitors, particularly tofacitinib, improve SAMHD1-related interferonopathy across diverse paediatric phenotypes.

## Introduction

Biallelic loss-of-function mutations in the SAMHD1 gene have been implicated in Aicardi-Goutières syndrome type 5 (AGS5), a paradigmatic example of type I interferon-mediated neuroinflammatory disorders predominantly targeting the central nervous system [1, 2]. AGS5 usually manifests in early infancy with features such as developmental delay or regression, cerebral calcifications, diffuse white matter abnormalities consistent with leukodystrophy and persistent systemic inflammation. The SAMHD1 gene encodes a deoxynucleoside triphosphate (dNTP) hydrolase, which serves a dual role: maintaining intracellular dNTP homeostasis and suppressing innate immune responses by preventing inappropriate activation of cytosolic nucleic acid sensors [2, 3].

Loss of functional SAMHD1 activity results in intracellular accumulation of endogenous nucleic acids, particularly DNA species. These nucleic acids can aberrantly engage cytosolic sensors such as cGAS–STING, leading to persistent type I interferon activation and chronic tissue inflammation [3]. Although Aicardi-Goutières syndrome type 5 (AGS5) has historically been characterized by hallmark neuroimaging findings and central nervous system involvement, recent reports have expanded the phenotypic landscape of SAMHD1 deficiency to include diverse systemic, cutaneous and rheumatologic manifestations [4, 5]. Beyond the central nervous system, involvement of the skin, musculoskeletal system and vasculature has been increasingly reported. Phenotypes resembling dermatomyositis, panniculitis, systemic lupus erythematosus and even vasculitic processes have been described, often without neuroimaging abnormalities [5, 6].

In this case series, we present three paediatric patients carrying the same homozygous SAMHD1 mutation, each manifesting a distinct clinical phenotype. The first patient exhibited a chronic myopathy-dominant presentation, the second displayed cutaneous and neurologic features consistent with panniculitic vasculopathy and the third demonstrated a lupus-like connective tissue disease phenotype. Through this report, we aim to illustrate the phenotypic diversity of SAMHD1 deficiency and advocate for its inclusion in the differential diagnosis of childhood-onset inflammatory syndromes, even in the absence of typical AGS manifestations. This underscores the broad phenotypic spectrum observed in autoinflammatory diseases.

## Methods

Patients were identified retrospectively from the paediatric rheumatology and immunology clinics of our tertiary centre between 2017 and 2025. We reviewed children who presented with chronic, treatment-refractory systemic and/or cutaneous inflammation, panniculitis, calcinosis, vasculitic or lupus-/dermatomyositis-like features suggestive of a type I interferonopathy, after excluding more common acquired and monogenic autoinflammatory or autoimmune conditions. Detailed clinical, laboratory and radiological data were extracted from the electronic medical record. Disease course, treatment history and response to therapies were recorded longitudinally.

All patients underwent genetic analysis with whole-exome sequencing (WES), which identified the same homozygous missense variant in SAMHD1 (NM_015474.4: c.625G>A; p. Gly209Ser; rs121434516). This variant has been previously reported in association with Aicardi–Goutières syndrome and is listed as pathogenic in ClinVar. According to American College of Medical Genetics and Genomics (ACMG) guidelines, it was classified as pathogenic/likely pathogenic. No additional pathogenic or likely pathogenic variants were detected in SAMHD1 or in other genes associated with type I interferonopathies. The variant was confirmed by Sanger sequencing. Immunological evaluation included routine measurement of serum immunoglobulins (IgG, IgA, IgM, total IgE), complement components (C3, C4) and lymphocyte subset analysis by flow cytometry. Type I interferon-stimulated gene (ISG) signatures were not routinely available at our centre during the study period and could therefore not be systematically assessed in these patients. This study was approved by the Hacettepe University Non-Interventional Clinical Research Ethics Committee (Approval No: SBA 25/799). Written informed consent was obtained from the parents/guardians of all participants.

## Case presentations

### Patient 1: a myopathy-dominant phenotype

A 10.5-year-old girl was evaluated for a two-year history of progressive difficulty in squatting, limited shoulder mobility when combing her hair and gait abnormality characterized by dragging of her right foot in a circumductive pattern. Despite the absence of overt joint swelling or inflammation, she reported mild morning stiffness lasting 3–5 min. Physical examination revealed fixed flexion contractures of the elbows, wrists and knees, along with bilateral shoulder restriction and positive Gowers’ sign. Dermatologic findings included heliotrope-like discoloration around the eyes, intermittent palmar erythema and episodes of non-painful oral aphthae.

There was no history of malar rash, photosensitivity, hair loss or genital ulcers. Muscle enzymes were mildly elevated (CK 169 U/l) and lactate dehydrogenase (LDH) was persistently raised (up to 618 U/l). ANA and anti-Scl-70 antibodies were positive; C3 and C4 were within normal limits. Immunological evaluation showed normal immunoglobulin and complement levels, with balanced lymphocyte subsets (CD3, CD4, CD8, NK and B cells within expected ranges). Notably, total IgE was elevated (1446 kIU/l). Cranial MRI revealed no intracranial calcifications. Thigh MRI showed bilateral atrophic changes compatible with chronic myositis. Electromyography (EMG) did not reveal evidence of active myopathic or neuropathic changes. Genetic analysis confirmed a homozygous missense variant in SAMHD1 (c.625G>A, p. Gly209Ser), evaluated as ‘likely pathogenic’ according to ACMG guidelines and previously reported in association with interferonopathies. No additional pathogenic variants were identified. She was initially treated with tofacitinib and experienced partial improvement, although the treatment course during the early phase was not optimal. After re-establishing a stable and consistent treatment regimen, a clearer improvement in joint mobility and functional performance was observed. Under tofacitinib therapy, her functional capacity progressively improved; she became able to rise from the floor without using her hands and could climb stairs without support. Her Childhood Myositis Assessment Scale (CMAS) score increased from 34 to 45 points over four months, indicating meaningful improvement in proximal muscle strength and endurance. Serum LDH levels decreased from 612 U/l to 389 U/l, supporting the objective clinical and biochemical response. The overall phenotype suggested a chronic inflammatory myopathy distinct from classical AGS.

### Patient 2: a CNS dominant phenotype with panniculitis

A male infant presented with erythematous nodular lesions over the anterior tibial surfaces and dorsum of the feet, which progressed to panniculitis-like plaques extending to the knees. Concurrent swelling was noted in the dorsal aspect of the hands and forearms, without frank arthritis. Low-grade fevers and mouth ulcers occurred intermittently. Skin biopsy confirmed mixed panniculitis. Brain MRI performed due to developmental delay revealed subcortical and basal ganglia calcifications, hypomyelination and cerebral volume loss. He was the fourth child of consanguineous (third-degree) parents. His father had a history of familial Mediterranean fever. Over time, he developed progressive digital contractures and calcified nodules. Whole-exome sequencing identified the same homozygous SAMHD1 mutation (c.625G>A, p. Gly209Ser), previously reported as pathogenic in association with interferonopathies. No other variants of clinical significance were detected. Tofacitinib (2 × 3.5 mg) was started with notable improvement in systemic inflammation and skin lesions. The number and tenderness of panniculitic nodules decreased markedly, with active nodules reducing from six to two within three months of therapy. Parents also reported that flare duration shortened and daily activity tolerance increased. However, relapses occurred during interruptions or dose reductions. Adjunctive corticosteroids and intravenous immunoglobulin (IVIG) were used during flare episodes. Neurologically, he maintained stable function with no seizure activity. Immunological studies were largely unremarkable, with normal complement and lymphocyte profiles, except for mildly reduced memory B-cell subsets. This constellation of panniculitis, cerebral calcifications, systemic inflammation and chronic progression strongly supported the diagnosis of interferonopathy.

### Patient 3: a lupus-like/connective tissue disease phenotype

A female patient initially presented at six years of age with recurrent facial rash, joint discomfort and photosensitivity. Skin involvement fluctuated but never completely resolved, characterized by erythematous plaques on the face, limbs and trunk. She also reported morning stiffness, finger swelling and livedo-like discoloration in cold environments. A skin biopsy from the thigh revealed histopathologic findings suggestive of tumid lupus *vs* granuloma annulare. Despite the absence of significant fever episodes, the chronicity and distribution of the rash raised concerns for autoimmune pathology. Brain MRI showed cerebellar telangiectasia and developmental venous anomalies, but no calcifications. Ophthalmologic examination revealed mild optic disc pallor. She gradually developed digital calcinosis, contractures and decreased range of motion in multiple joints. She had no history of seizures or overt neurologic regression. Multiple immunomodulatory therapies were trialled: tofacitinib (with initial improvement), baricitinib (limited effect) and later ruxolitinib due to ulcerative lesions on digits and buttocks. IVIG was added to address refractory calcinosis and dermatomyositis-like features. ANA and myositis antibody panels were intermittently tested and were mostly negative, with results remaining largely inconclusive. Genetic testing confirmed the same homozygous SAMHD1 missense variant (c.625G>A, p. Gly209Ser), documented in interferonopathy cohorts and classified as pathogenic by ACMG criteria. Immunological assessment showed IgG 13.9 g/l, and IgA 2.19 g/l within normal limits, while other parameters were unremarkable. In addition, because of the presence of telangiectasia, alpha-fetoprotein level was measured and found to be close to the upper limit of normal.

Her disease course was relapsing-remitting, and tofacitinib provided the most consistent benefit. During stable tofacitinib therapy, digital and buttock ulcerations became infrequent, with only one mild episode occurring over six months. Existing ulcers healed more rapidly, and cold-induced livedoid discoloration diminished. Calcinosis deposits softened with reduced perilesional inflammation, contributing to improved joint mobility and hand function. The clinical picture suggested a unique overlap between autoinflammatory interferonopathy and connective tissue disease. To contextualize the phenotypic diversity observed in our cohort, we have revised and expanded Table 1 to include a comparative overview of our three patients together with previously reported paediatric SAMHD1/AGS cases.

**Table 1.** Comparative analysis of our three patients with previously reported paediatric SAMHD1/AGS cases in the literature

Feature	Our patient 1	Our patient 2	Our patient 3	Previously reported SAMHD1/AGS cases
**Genotype**	c.625G>A (homozygous)	c.625G>A (homozygous)	c.625G>A (homozygous)	Biallelic pathogenic variants common [7–9]
**Age at onset**	8 years	Infancy	6 years	Typically infancy, but late-onset described [8]
**Dominant phenotype**	Myopathy-dominant	Panniculitis + CNS	Lupus-like/CTD	Broad phenotype: CNS, lupus-like, panniculitis, vasculopathy [10–12]
**Neurological findings**	MRI normal	Calcifications	Vascular anomalies	Classical CNS findings: intracranial calcifications, leukodystrophy [7, 8, 13]
**Cutaneous findings**	Mild heliotrope	Panniculitis	Lupus-like rash, calcinosis	Skin involvement: chilblain, panniculitis, ulcerations [7–9, 11]
**Calcinosis**	Mild	Digital + subcutaneous	Extensive	Calcinosis reported in interferonopathies [13]
**Autoimmune features**	ANA+, anti–Scl-70+	Negative	Biopsy: tumid lupus *vs* GA	Autoimmunity (SLE/Sjögren) frequently reported in SAMHD1 cohorts [12, 14]
**Muscle involvement**	Chronic myositis	Contractures	Joint stiffness	Myositis-like phenotype described [13]
**Neuroimaging**	Normal	Classical AGS	Non-classical	MRI-negative interferonopathy also recognized [14]
**Immunological findings**	IgE ↑	Mild B-memory ↓	Normal IgA/IgG	Typically normal IgG/IgA/IgM [15]
**Interferon signature**	Not available	Not available	Not available	Consistently elevated in published cohorts [7]
**Treatment**	Tofacitinib	Tofacitinib + IVIG + steroids	Tofacitinib > Ruxolitinib > Baricitinib	JAK inhibitors used in SAMHD1/AGS [11, 14]
**Response to JAK-i**	Partial → sustained	Improvement, flares if interrupted	Best with tofacitinib	Variable responses, but improvement reported [11, 14]
**Overall phenotype category**	Atypical interferonopathy	Classical AGS-like	Autoimmune/CTD-like	Spectrum: classical AGS → lupus-like → vasculopathic → myositis-like [8, 9, 12, 13]

Despite carrying the identical pathogenic variant, the patients demonstrated markedly distinct clinical phenotypes, ranging from chronic inflammatory myopathy and panniculitis with cerebral calcifications to a lupus-like systemic inflammatory syndrome. All three patients were treated with JAK inhibitors but exhibited varying degrees of therapeutic response.

ANA, antinuclear antibody; CNS, central nervous system; F, female; GA, granuloma annulare; LDH, lactate dehydrogenase; M, male.

## Discussion

This case series highlights the remarkable phenotypic heterogeneity observed in individuals with biallelic SAMHD1 mutations, even when carrying the same homozygous pathogenic variant. All patients had presented to our centre and all were Turkish. While Aicardi-Goutières syndrome type 5 (AGS5) is classically typified by neuroinflammatory findings and intracranial calcifications, the clinical manifestations in our cohort extended beyond these canonical features. The first patient exhibited a myopathy-dominant phenotype with elevated serum LDH and radiologic evidence of chronic muscle inflammation in the absence of any central nervous system involvement, mimicking juvenile dermatomyositis. The second patient had a more expected phenotype, presenting with classical interferonopathy characteristics, including basal ganglia calcifications, lobular panniculitis and systemic inflammation. The third patient displayed a phenotype reminiscent of paediatric-onset systemic lupus erythematosus and juvenile systemic sclerosis, characterized by connective tissue involvement, subcutaneous calcinosis and features suggestive of peripheral vasculopathy such as Raynaud-like episodes. Immunological investigations were largely unremarkable, with normal serum immunoglobulins and lymphocyte subsets across patients, except for a mild reduction in memory B cells in patient 2. These findings support the concept that SAMHD1 deficiency manifests as an interferonopathy rather than a classical immunodeficiency. Importantly, the clinical heterogeneity observed among patients carrying the identical homozygous variant points to the influence of additional regulatory factors, including epigenetic mechanisms, individual immune responsiveness and environmental exposures. Such modulatory factors may help explain the divergent clinical trajectories observed among genetically identical patients [16]. In patient 3, alpha-fetoprotein was measured due to the presence of cerebellar telangiectasia and was found at the upper limit of normal, although no additional clinical or laboratory features supported a diagnosis of ataxia–telangiectasia.

Despite carrying the identical homozygous SAMHD1 variant (c.625G>A, p. Gly209Ser), our patients exhibited strikingly divergent clinical phenotypes, underscoring the role of epigenetic factors, environmental triggers and individual immune responsiveness. SAMHD1 maintains immune homeostasis by preventing inappropriate cytosolic nucleic acid sensing and dampening downstream type I interferon responses; loss-of-function mutations disrupt this balance, leading to persistent interferon signalling and the development of autoinflammatory and autoimmune manifestations. In recent years, both clinical and experimental studies have provided important insights into how SAMHD1 deficiency drives type I interferon dysregulation. For example, Kretschmer *et al.* demonstrated that SAMHD1 deficiency leads to imbalanced dNTP pools, genome instability and chronic DNA damage signalling, which in turn activates type I interferon pathways and promotes autoimmunity [17]. Furthermore, Abe *et al.* in a nationwide Japanese cohort, reported that all AGS patients harbouring SAMHD1 mutations developed autoimmune diseases, including systemic lupus erythematosus and, notably, the first described case of Sjögren’s syndrome, highlighting the particularly strong association of SAMHD1 with autoimmunity [18]. This mechanism underlies the pathogenesis of Aicardi–Goutières syndrome type 5, as initially described by Rice *et al.* in human cohorts demonstrating a robust interferon-stimulated gene signature [2].

In parallel, Behrendt *et al.* provided supportive evidence in mouse models, showing that murine Samhd1 suppresses a spontaneous cell-intrinsic antiviral response, further underscoring its role in innate immune regulation [19]. Using functional genomics and iPSC-derived models, Coquel *et al.* revealed that SAMHD1 preserves genome integrity by acting at stalled replication forks and limiting interferon production, further supporting its essential role in safeguarding both nucleic acid metabolism and immune homeostasis [3].

This inter-individual variability raises important questions regarding the influence of modifier genes, epigenetic factors and environmental triggers on disease expression. It also emphasizes the diagnostic challenge posed by non-classical presentations of interferonopathies, particularly in paediatric patients with multisystem inflammatory features.

A limitation of our report is the lack of longitudinal type I interferon signature measurements, which would have allowed us to correlate clinical responses more directly with suppression of interferon-stimulated gene expression. Nonetheless, the observed clinical improvement under JAK inhibition and the underlying genetic diagnosis strongly support an interferon-driven pathogenesis in these children.

From a therapeutic standpoint, Janus kinase (JAK) inhibitors such as tofacitinib and baricitinib have shown notable efficacy in ameliorating type I interferon-mediated clinical manifestations in interferonopathies, including Aicardi–Goutières syndrome (AGS) [20, 21]. The features of our patients also improved with JAK inhibitors. In a cohort of patients treated with JAK inhibitors, rapid resolution or improvement of cutaneous lesions and systemic inflammatory signs was documented, alongside normalization of interferon scores and inflammatory markers [21]. In our series, continuous therapy was crucial. Importantly, treatment interruptions were consistently associated with disease flares, emphasizing the need for continuous JAK inhibitor therapy in SAMHD1-associated interferonopathies. Among JAK inhibitors, tofacitinib provided the most consistent benefit, while baricitinib and ruxolitinib yielded variable responses. Although our numbers are small, one possible explanation is the differential JAK selectivity and tissue distribution of these agents. Tofacitinib predominantly targets JAK1 and JAK3, thereby efficiently blocking signalling downstream of multiple γ-chain cytokines and type I interferons, which may be particularly relevant in SAMHD1-driven disease. In contrast, baricitinib and ruxolitinib mainly inhibit JAK1/JAK2, with potentially different effects on cytokine networks and clinical manifestations. Furthermore, individual differences in drug exposure, adherence and organ involvement may contribute to the heterogeneous responses observed in our patients. However, continuous treatment appears necessary, as disease exacerbations have been observed following interruptions. In certain refractory cases, supplemental therapies such as IVIG and systemic corticosteroids were required [1]. Taken together, these observations highlight the clinical importance of early recognition and prompt initiation of targeted immunomodulation in SAMHD1-associated interferonopathies, especially given their phenotypic variability.

## Conclusion

Our findings expand the phenotypic spectrum of SAMHD1 deficiency and emphasize that this diagnosis should be considered in children with unexplained systemic inflammation, even in the absence of classical neuroimaging features of AGS. SAMHD1 deficiency may manifest with isolated myositis, panniculitis or lupus-like disease, underscoring the importance of a phenotype-driven rather than genotype-based diagnostic approach. Timely recognition and early genetic testing for interferonopathies, including atypical SAMHD1-related syndromes, are therefore essential to guide targeted immunomodulatory therapy. Importantly, therapeutic responses to JAK inhibitors across divergent phenotypes support a shared interferon-driven pathogenic mechanism, and sustained therapy appears crucial, as treatment discontinuation frequently triggered relapses. Early intervention with such targeted therapies may not only relieve symptoms but also modify disease trajectory. As precision medicine advances, unravelling the interplay of genetic, epigenetic and immunological factors will be critical to optimizing outcomes for affected patients. From a practical standpoint, our observations suggest several clinical scenarios in which genetic evaluation for SAMHD1 deficiency should be considered in children with seemingly common autoimmune diseases such as juvenile dermatomyositis or systemic lupus erythematosus. These include: (i) early-onset, treatment-refractory myositis or panniculitis with prominent calcinosis; (ii) lupus- or dermatomyositis-like phenotypes accompanied by digital vasculopathy, livedo, or extensive subcutaneous calcifications; (iii) the coexistence of neuroimaging abnormalities (e.g. basal ganglia calcifications or unexplained white-matter changes) with chronic systemic inflammation; and (iv) patients from consanguineous families in whom multiple autoimmune or autoinflammatory features cluster. In such contexts, early referral for whole-exome sequencing or targeted Sanger sequencing of SAMHD1 may facilitate timely diagnosis and initiation of interferon-targeted therapy.

## Data Availability

The data underlying this article cannot be shared publicly due to patient privacy and confidentiality restrictions. The data will be shared on reasonable request to the corresponding author, subject to institutional and ethical approval.
